# Penetration Behavior of High-Density Reactive Material Liner Shaped Charge

**DOI:** 10.3390/ma12213486

**Published:** 2019-10-24

**Authors:** Huanguo Guo, Jianwen Xie, Haifu Wang, Qingbo Yu, Yuanfeng Zheng

**Affiliations:** State Key Laboratory of Explosion Science and Technology, Beijing Institute of Technology, Beijing 100081, China; 3120160127@bit.edu.cn (H.G.); 3120180260@bit.edu.cn (J.X.); wanghf@bit.edu.cn (H.W.); yuqb@bit.edu.cn (Q.Y.)

**Keywords:** shaped charge, reactive materials, high-density liner, jet formation, penetration behavior

## Abstract

The traditional polytetrafluoroethylene (PTFE)/Al reactive material liner shaped charge generally produces insufficient penetration depth, although it enlarges the penetration hole diameter by chemical energy release inside the penetration crater. As such, a novel high-density reactive material liner based on the PTFE matrix was fabricated, and the corresponding penetration performance was investigated. Firstly, the PTFE/W/Cu/Pb high-density reactive material liner was fabricated via a cold pressing/sintering process. Then, jet formation and penetration behaviors at different standoffs were studied by pulse X-ray and static experiments, respectively. The X-ray results showed that the PTFE/W/Cu/Pb high-density reactive material liner forms an excellent reactive jet penetrator, and the static experimental results demonstrated that the penetration depth of this high-density reactive jet increased firstly and then decreased by increasing the standoff. When the standoff was 1.5 CD (charge diameter), the penetration depth of this reactive jet reached 2.82 CD, which was significantly higher than that of the traditional PTFE/Al reactive jet. Moreover, compared with the conventional metal copper jet penetrating steel plates, the entrance hole diameter caused by this high-density reactive jet improved 29.2% at the same standoff. Lastly, the chemical reaction characteristics of PTFE/W/Cu/Pb reactive materials were analyzed, and a semi-empirical penetration model of the high-density reactive jet was established based on the quasi-steady ideal incompressible fluid dynamics theory.

## 1. Introduction

Reactive material liner is a kind of integrated solid energetic liner prepared by filling metal powders in a high polymer binder, typically such as polytetrafluoroethylene (PTFE)/Al and PTFE/Cu, and then fabricating by a cold pressing and sintering process [1,2]. Generally, the reactive material liner under the shaped charge effects can form a reactive jet with high velocity to penetrate the target and then release its violent chemical energy inside the penetration crater, resulting in extremely large amounts of damage to concrete or geologic material targets [3]. Furthermore, an explosively formed projectile (EFP) made of reactive materials can also achieve greater behind-armor effects by incorporating the defeat mechanisms of kinetic energy and chemical energy [4].

Due to excellent damage performances and potential applications in military, the reactive material liner and the corresponding shaped charge technique have received much attention extensively over the past twenty years. The energy release characteristics of reactive material liners with different formulations were analyzed by using the JAGUAR thermochemical equation, and the coupled defeat effects of kinetic energy and chemical energy for reactive material liner shaped charges against desired targets were preliminarily conducted by experiments [3]. Daniels et al. studied the reactive jet formation behavior by simulation and further conducted experiments on the damage behavior of large-caliber reactive liner shaped charges against typical targets, such as concrete culvert and a thick asphalt roadway [5]. Compared with the single metal copper jet and aluminum jet, the PTFE/Al reactive jet produced enhanced structural damage to the multi-layered concrete target, but the experiments indicated its penetration depth was low [6,7]. The behind-armor overpressure characteristics of the PTFE/Al reactive jet were investigated, and a semi-empirical analysis model of the behind-armor overpressure was established by introducing a reaction delay time of reactive materials [8]. The penetration behaviors of PTFE/Al reactive jets against thick steel plates under different standoffs were studied, and the experiments showed that the fragmentation effects of penetrated steel plates were produced at the standoff of 0.5, 1.0, and 1.5 CD (charge diameter), whereas the penetration depths were only approximately 1.0 CD [9,10]. Yin et al. and Wang et al. compared and analyzed the penetration performance of a PTFE jet and PTFE/Cu jet impacting shell charge and main armor by simulations and experiments combined, and the results verified that the penetration capability of the PTFE/Cu jet increased to some extent [2,11]. In addition to PTFE-based reactive material liners, metal powders reactive liners have been widely studied in recent years. Claridge et al. studied the jet formation behavior of Ni/Al reactive material liner shaped charge based on an X-ray experiment [12]. Sun et al. compared the penetration performance between Ni-Al and Cu-Ni-Al reactive material liner shaped charges, and the experiments showed that the addition of Cu powders not only increased the average penetration hole diameter, but also improved the average penetration depth [13]. J. Evers et al. presented the melting temperature of W and Pb metals, and further studied the high density of the system ZrW_2_ reactive material and its strong exothermic reaction characteristics, providing a novel kind of reactive material for warhead applications [14].

Based on the studies mentioned above, it can be seen that mainly significant progresses have been made in the formulation and preparation of reactive material liners, the formation behaviors of reactive jets, and the penetration enhanced behaviors of reactive material liner shaped charges. However, the aforementioned studies mostly focused on low-density reactive material liners, and their insufficient penetration depths significantly limit the application of reactive materials on shaped charge warheads. As such, this paper firstly proposes a high-density reactive material liner based on the PTFE/W/Cu/Pb formulation system. An X-ray experiment was conducted to analyze the formation behavior of the high-density reactive jet, and penetration experiments were carried out to investigate the penetration performance of the high-density reactive jet under different standoffs. Finally, the reaction mechanism of PTFE/W/Cu/Pb reactive materials was discussed, and a theoretical penetration model was established by introducing the reaction delay time of reactive materials.

## 2. High-Density Reactive Material Liners

### 2.1. Reactive Liner Materials

The high-density reactive materials of the shaped charge liner were formed by mixing three kinds of metal powders—tungsten (W), copper (Cu), and plumbum (Pb)—into the PTFE matrix. The reactive materials focusing on PTFE/W/Cu/Pb composites have a mass ratio of 5/20/45/30, considering both density and stoichiometry. The theoretical density of the reactive materials was 8.90 g/cm^3^. The average particle size of the PTFE powder used in the formulation system was 40 nm. Additionally, the average particle sizes of W, Cu, and Pb powders were 74 μm, 83 μm, and 74 μm, respectively. Actually, PTFE powder is characterized by its excellent formability and acts as a binder for the composites. More importantly, the PTFE decomposes during the penetration process under shock loadings, releasing large amounts of fluoride gases, which are used as oxidizing agents for the reaction system. W powder is chosen as an indispensable constituent for its high density. Moreover, Cu and Pb powders are added to the composites to mainly improve the jet continuity during the formation process based on their excellent ductility, and both of them react vigorously and exothermically with the fluoride decomposed and released by PTFE. In particular, the Pb also gasifies during the penetration process to produce extra damage to the target.

### 2.2. Reactive Material Liner Preparation

**Figure 1:**
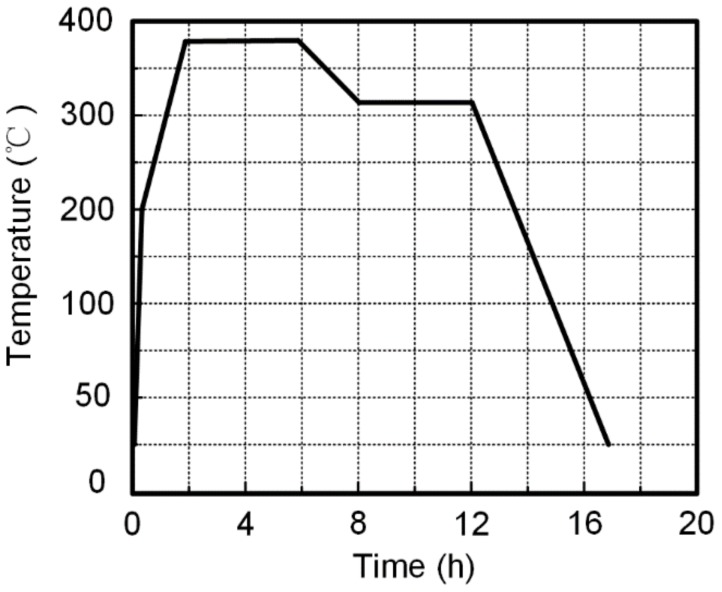
The sintering cycle of polytetrafluoroethylene (PTFE)/W/Cu/Pb reactive material liner samples.

The preparation process for the PTFE/W/Cu/Pb reactive material liner includes the following steps [15,16]:

(1) First, the powders were mixed by a planetary mill machine for 3 h, with a small amount of absolute alcohol as a medium. Then, the powders were dried at 82 °C in a vacuum drying oven for 24 h approximately.
(2) Second, 45.0 g of well-mixed powder was weighed and placed in a pressing mold. A cold pressing pressure of 450 MPa was maintained for 30 s. The pressed liner samples were then relaxed at ambient pressure and temperature for 24 h in order to remove trapped air and residual stress.
(3) Third, the pressed liner samples were then sintered in a nitrogen atmosphere with a maximum temperature of 380 °C. Figure 1 shows the temperature history of the sintering cycle, which can be described as follows: the oven temperature was raised to 200 °C at a rate of about 10 °C/min, and then upgraded to 380 °C at a rate of about 2 °C/min. The samples were held at 380 °C for 4 h, then the temperature was reduced at a rate of about 0.5 °C/min to 315 °C and maintained for 4 h. The sintered liner samples were then cooled to ambient temperature at an average cooling rate of 1 °C/min.

**Figure 2:**
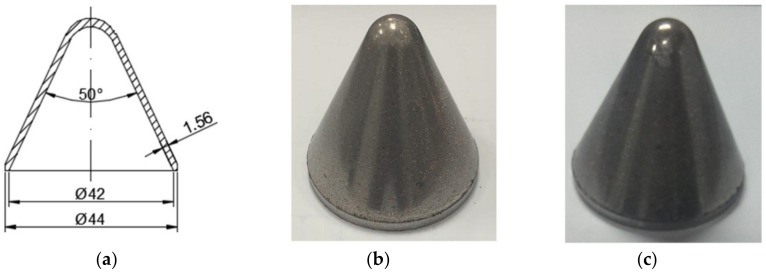
Sketch and photographs of PTFE/W/Cu/Pb liners at different preparation stages: (**a**) sketch; (**b**) pressed samples; (**c**) sintered samples.

Sketch and photographs of PTFE/W/Cu/Pb liners at different preparation stages are shown in Figure 2. It should be mentioned that the actual density of samples is about 8.51 g/cm^3^, indicating a porosity of 4.4%.

## 3. X-Ray Experiments

Different from the traditional inert metal liner, the PTFE/W/Cu/Pb high-density reactive material liner is initiated and then a chemical reaction under the detonation effects of the shaped charge occurs, which may have a significant influence on the jet formation. As such, the jet formation behavior was studied and stressed in this section by X-ray experiments.

### 3.1. High-Density Reactive Material Liner Shaped Charge

**Figure 3:**
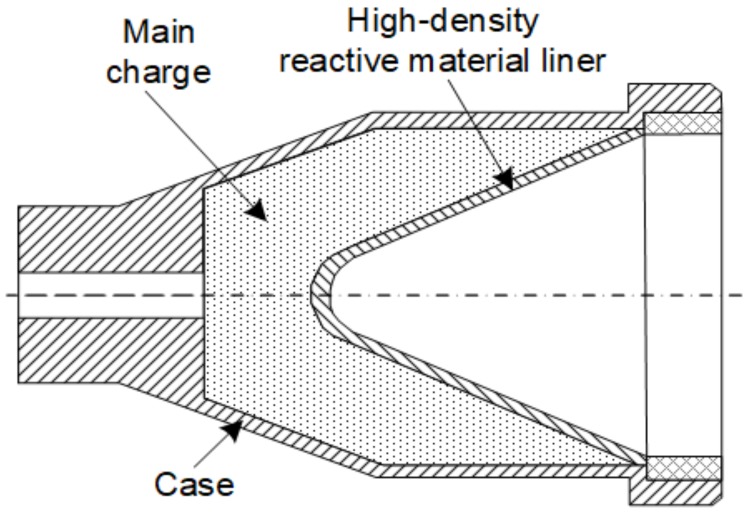
Diagram of high-density reactive material liner shaped charge.

The high-density reactive material liner shaped charge, as illustrated in Figure 3, mainly included a main charge, a high-density reactive material liner, and a case. The main charge was obtained by pressing JH-2 explosive in a mold with a pressure of 200 MPa. The density, diameter, and height of the main charge are 1.70 g/cm^3^, 44 mm, and 60 mm, respectively. The 2-mm thick case was machined by LY12 aluminum.

### 3.2. X-ray Experimental Setup

**Figure 4:**
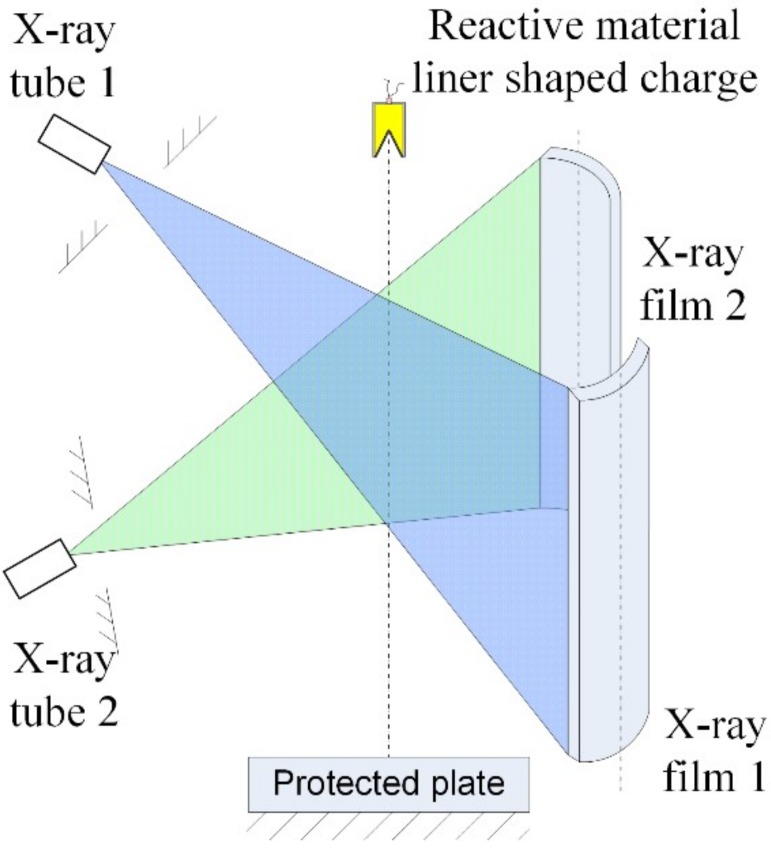
Experimental principle of X-ray.

The pulse X-ray test technique was used to observe the jet formation behavior. In the testing process, two X-ray tubes were used in different positions, and the two rays intersected with the axis of the shaped charge with a certain angle. Different ray emission delay times were set for the two ray tubes, thus two appearances of the jet at different times were obtained on the negative films. The experimental principle of the X-ray is illustrated in Figure 4.

### 3.3. X-ray Results

**Figure 5:**
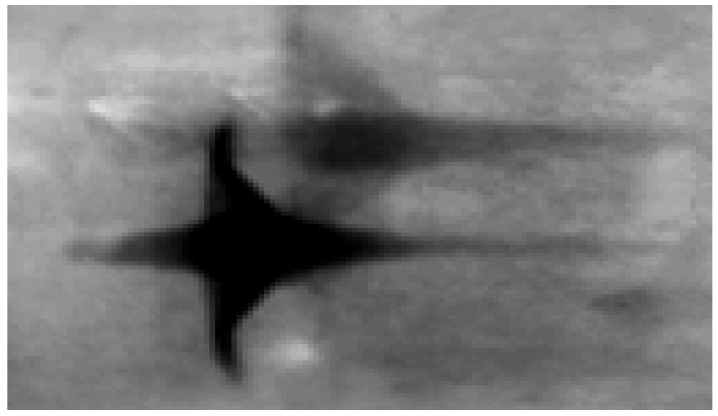
X-ray results of the high-density reactive jet formation.

The jet formation results of the high-density reactive material liner shaped charge are shown in Figure 5. The jet pictures were taken at different time delays: 24 μs and 28 μs, counting from the time of the main charge initiation. From the X-ray pictures of the shape of reactive jets, the length of reactive jets at different times can be evaluated, and then the average jet tip velocities at different times also can be calculated based on the jet position at the given moments. The results revealed that this class of PTFE/W/Cu/Pb reactive material liner could form a reactive jet with excellent performance, including the distinct jet outline, clear jet tip and rear, and satisfied jet continuity. The radiographs in Figure 5 show the reactive jet formed after 24 μs and 28 μs from initiation. The positions of the jets reached are approximately 1.5 CD and 2.0 CD of standoff, and the corresponding jet tip velocities are approximately 6213 m/s and 6170 m/s, respectively. Furthermore, the clear appearances of the jet indicate that there is no violent chemical reaction during the jet formation process.

## 4. Penetration Experiments

### 4.1. Experimental Setup

**Figure 6:**
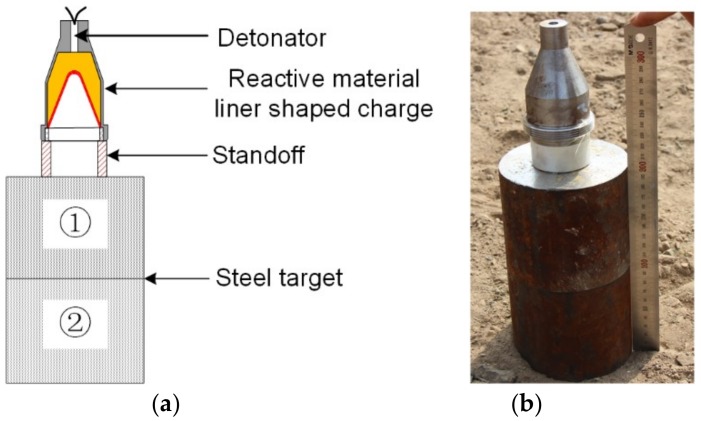
Schematic and photograph of the experimental setup: (**a**) schematic; (**b**) setup.

The experimental setup of the PTFE/W/Cu/Pb reactive material liner shaped charges against steel targets is depicted in Figure 6. It mainly consists of a detonator, a high-density reactive material liner shaped charge, a standoff, and a steel target. It should be noted that the reactive material liner shaped charges used in penetration experiments are the same as that employed in X-ray experiments. The detonator was fixed in the center of the main charge bottom to guarantee a center point initiation. To investigate the effect of the standoff on the penetration performance of the PTFE/W/Cu/Pb reactive jets, nylon standoff tubes of four different heights (0.5, 1.0, 1.5, and 2.0 CD) were used to adjust the heights from the bottom of the charge to the steel target. To facilitate the measurement of the penetration depth and observation of the follow-through behavior of the reactive materials, the steel target was composed of two 100-mm thick #45 steel plates.

### 4.2. Penetration Performance

**Figure 7:**
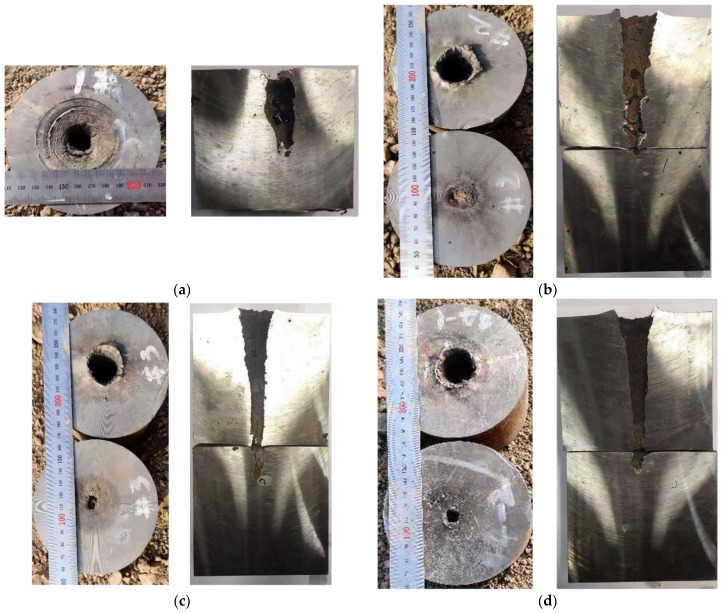
Experimental results of high-density reactive jets against steel targets under different standoffs (on the left is the entrance hole view of the steel plates; on the right is a section view of the steel plates): (**a**) H = 0.5 CD; (**b**) H = 1.0 CD; (**c**) H = 1.5 CD; (**d**) H = 2.0 CD.

The experimental results of the high-density reactive liner shaped charges against steel targets are summarized in Table 1. The typical experimental photographs of high-density reactive jets penetrating steel targets under different standoffs are shown in Figure 7, which indicates that the penetration performance of these high-density reactive jets is influenced significantly by the standoff. The experimental results show that with the standoff increasing from 0.5 CD to 2.0 CD, the penetration depth first increases and then decreases. As can be seen from the section view of Figure 7, when the standoff is 0.5 CD, the high-density reactive jet fails to perforate the first steel plate, whereas the jets can perforate the 100-mm thick steel plates at the standoffs of 1.0, 1.5, and 2.0 CD. In particular, the largest penetration depth is approximately 2.82 CD when the reactive jet against steel target is at a standoff of 1.5 CD. 

According to Table 1, the penetration hole diameter also strongly depends on the standoff, and the entrance hole diameter of ① steel plate decreases gradually with an increasing standoff. When the standoff is 0.5 CD, the maximum entrance hole diameter is approximately 0.97 CD. For mechanism consideration, the smaller the standoff, the larger the tip velocity and diameter of the reactive jet, which will cause the penetration hole diameter to increase. At the same time, more reactive materials will enter the penetration hole at the smaller standoff, which will increase the pressure inside the penetration hole owing to the violent chemical reaction of reactive materials, resulting in a significant enhancement of the cratering expansion effect [10]. 

### 4.3. Penetration Comparison

**Figure 8:**
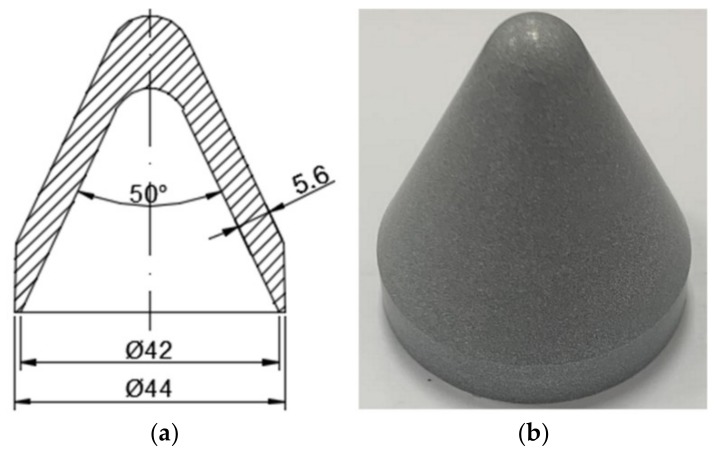
Structure and specimen of the PTFE/Al reactive liner: (**a**) structure; (**b**) liner specimen.

**Figure 9:**
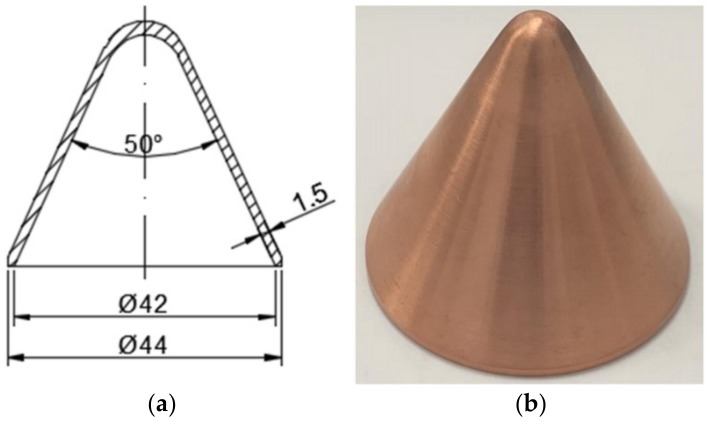
Structure and specimen of the metal copper liner: (**a**) structure; (**b**) liner specimen.

**Figure 10:**
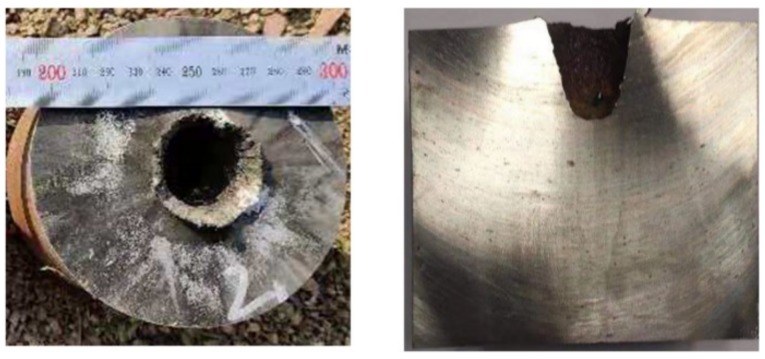
Experimental results of the PTFE/Al reactive jet against the steel target (on the left is the entrance hole view of the steel plates; on the right is the section view of the steel plates).

**Figure 11:**
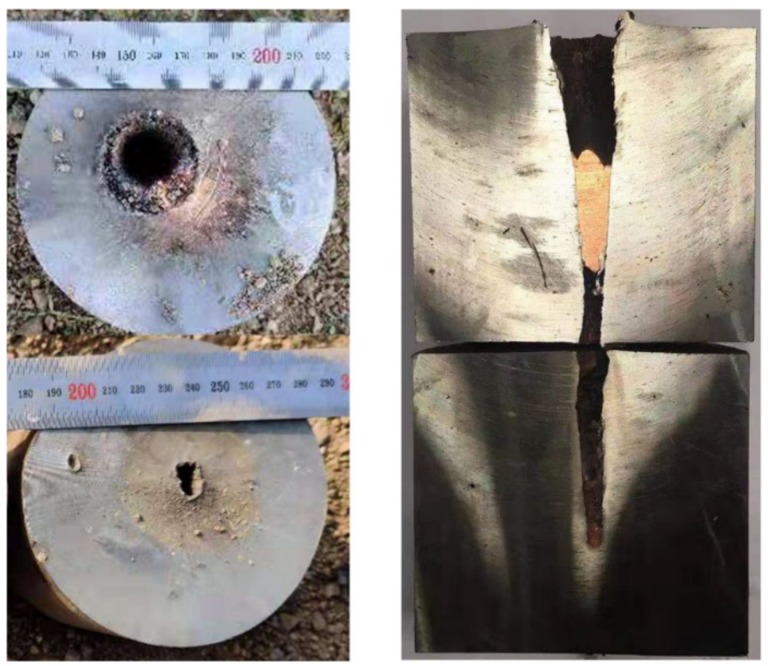
Experimental results of the copper jet against the steel target (on the left is the entrance hole view of the steel plates; on the right is the section view of the steel plates).

In this section, experiments of the PTFE/Al reactive material liner shaped charge and the metal copper liner shaped charge against steel targets were conducted for comparison. The wall thickness of the liner was adjusted so that the mass of the PTFE/Al reactive liner and metal copper liner would be the same as that of the PTFE/W/Cu/Pb high-density reactive material liner due to the different density for three kinds of liners, as listed in Table 2 [17]. The structure and specimen of the PTFE/Al reactive liner and the metal copper liner are shown in Figure 8 and Figure 9, respectively. That is to say, although the wall thicknesses of these three kinds of liners were different, the other conditions of the three shaped charges were consistent to better compare the penetration performance. The standoffs were all chosen as 1.5 CD when the different jets penetrating steel targets. Figure 10 and Figure 11 show the experimental results of the PTFE/Al reactive jet and the copper jet against steel targets, respectively. Table 2 presents the comparison of the penetration depth and entrance hole diameter of steel targets impacted by different jets.

As presented in Table 2, the penetration depth of the PTFE/W/Cu/Pb reactive jet is significantly greater than that of the PTFE/Al reactive jet, which is mainly due to the higher density of the PTFE/W/Cu/Pb reactive materials. However, the penetration depth of the PTFE/W/Cu/Pb reactive jet is smaller than that of the metal copper jet. The main reasons are as follows: (1) Compared with the density of the copper jet, the actual density of the PTFE/W/Cu/Pb reactive jet is slightly smaller; (2) the ductility of the jet formed by the pressed and sintered PTFE/W/Cu/Pb reactive material liner is inferior to that of the metal copper jet; and (3) the PTFE/W/Cu/Pb reactive jet will incur a violent chemical reaction during the penetration process, which will lead to an early termination of the penetration process [17]. In addition, a comparison among Figure 7, Figure 10 and Figure 11 shows that there is a slug inside the penetration channel for the copper jet, whereas no slug is found for two kinds of reactive jets. Other than the deflagration reaction of PTFE/Al and PTFE/W/Cu/Pb reactive materials, another important reason is that the PTFE/Al and PTFE/W/Cu/Pb reactive material liners essentially belong to the powder liners, which causes the porous and grainy structure of the generated slug with low mechanical strength, also preventing the clogging of the penetration hole [18]. 

In terms of the penetration hole diameter, it increases in sequence of the copper jet, PTFE/W/Cu/Pb reactive jet, and PTFE/Al reactive jet. Compared with the copper jet, the entrance hole diameters of **①** steel plates produced by the PTFE/W/Cu/Pb reactive jet and PTFE/Al reactive jet increased by approximately 29.2% and 43.8%, respectively. For the mechanism analysis, the radial crater of the copper jet was jointly determined by the following two reasons: (1) When the copper jet penetrates the steel target at the intersection of the jet and the target, the jet undergoes a very high level of stress and the jet tip suffers a mushroom-shaped deformation, which means the jet particles consume their own energy to overcome the target resistance, thus producing a radial crater; (2) at the same time, the inertial forces generated by the flowing particles further expand this formed crater. However, for the PTFE/W/Cu/Pb and PTFE/Al reactive jets, in addition to the two reasons above, the reactive jet experiences a violent chemical reaction and produces significant overpressure within the formed crater, thus further improving the radial diameter. Furthermore, another two reasons should also be considered. On one hand, the liner thickness increases with decreasing the liner density, which causes the corresponding jet diameter to increase and eventually improve the initial penetration crater. On the other hand, the radial dispersion of the jets produced by the PTFE/W/Cu/Pb and PTFE/Al powder liners is larger than that of the monolithic copper liner [19], which leads to larger deformation of the initial penetration crater in the radial direction, eventually increasing the diameter of the entrance hole. In fact, compared with the PTFE/W/Cu/Pb reactive liner materials, the PTFE/Al reactive materials with the same mass release more chemical energy and gaseous products [17], which is a beneficial property to enhance the secondary expansion effect of the initial hole; thus, the penetration hole diameter of the PTFE/Al reactive jet is larger than that of the PTFE/W/Cu/Pb reactive jet.

## 5. Reaction Mechanism and Penetration Model

### 5.1. Reaction Mechanism

For the PTFE-based reactive material liners, the shaped charge not only effects jet formation, but also results in the temperature rise inside the reactive materials. When the temperature of reactive materials rises to 560 °C [15,20], the PTFE matrix experiences a violent decomposition reaction and releases C_2_F_4_ with strong oxidation. Subsequently, the metal powder particles of Cu and Pb react with C_2_F_4_, releasing large amounts of chemical energy and gaseous products. The chemical analysis results indicate that the main products include CuF, CuF_2_, PbF_2_, PbF, WC, and C. The possible chemical reactions of the PTFE/W/Cu/Pb reactive materials mainly include:

(−C_2_F_4_−) n → n C_2_F_4_ (1)

4Cu + C_2_F_4_ → 4CuF + 2C (2)

2Cu + C_2_F_4_ → 2CuF_2_+ 2C (3)

2Pb + C_2_F_4_ → 2PbF_2_ + 2C (4)

4Pb + C_2_F_4_ → 4PbF + 2C (5)

W + C → WC(6)

The masses of PbF_2_, CuF, PbF, and CuF_2_ in the preceding reaction formula decrease in sequence. The energy released by the chemical reaction is approximately 809 kJ/kg and the amount of gaseous product is approximately 40 L/kg.

Different from traditional energetic materials (such as explosives and propellants), the reaction mechanism of this high-density reactive material is more complex. In general, under the high-pressure of the shaped charge detonation, the reactive material liner collapses and compresses to form a reactive jet. It should be noted that the reactive material liner deforms significantly during the jet formation process, which may cause some hot spots within the reactive materials and a few local ignition reactions. However, the violent activation and deflagration reaction of the reactive materials not only require sufficient impact pressure, but also need a specific pressure duration. The transient peak pressure formed by the main charge detonation is extremely high, but the duration time is very short. Thus, the charge detonation can only lead to local ignition reactions adjacent to these hot spots. As time progresses, these local ignition reactions gradually spread via the heat conduction mechanism and finally develop into a violent deflagration reaction of the whole reactive jet. Since the heat conduction rate is lower than the jet formation and penetration rates, there is a time difference between the beginning of the reactive material liner subjected to the shock wave and the violent deflagration reaction, which is called the reaction delay time of the reactive jet (*τ*). Due to the existence of the reaction delay time, the high-density reactive material liner can form a jet and then penetrate the target. Therefore, it is generally believed that the deflagration reaction of the reactive jet during the formation and penetration process is extremely limited and can be ignored. When the time reaches the reaction time point, the high-density reactive jet will undergo a drastic deflagration reaction and produce large amounts of gaseous products and chemical energy within the penetration crater, resulting in the termination of the penetration process.

### 5.2. Penetration Model

**Figure 12:**
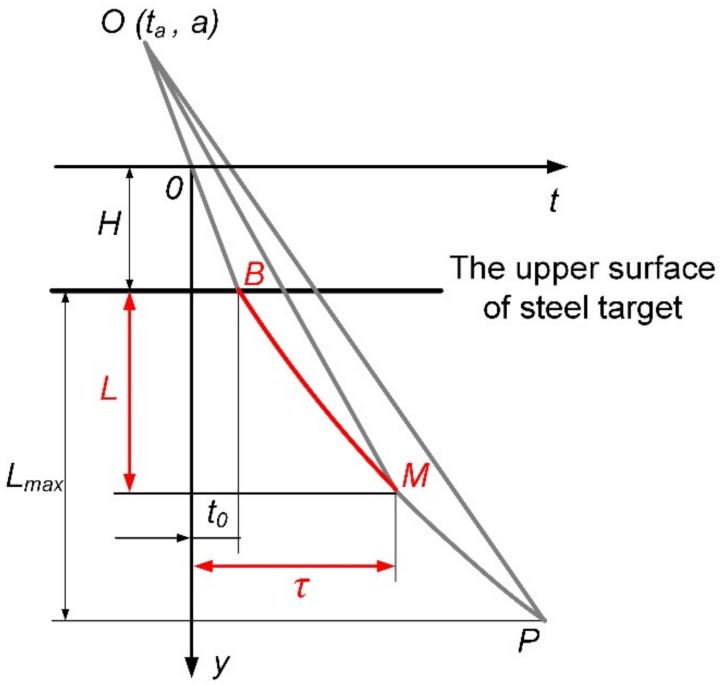
Theoretical model of penetration depth for a high-density reactive jet against a steel target.

According to studies on jet formation of X-ray and penetration experiments of high-density reactive material liner shaped charges against steel targets, it is assumed that the PTFE/W/Cu/Pb reactive jet is treated as inert within the reaction delay time, and all reactive materials simultaneously and instantaneously incur chemical reactions when the time reaches the reaction delay time of the reactive jet. Moreover, the strength effect of the target plate is ignored during the penetration process. Based on the quasi-steady ideal incompressible fluid theory [21,22], a semi-empirical analysis model can be established for the penetration behavior of the high-density reactive jet against a steel target, as depicted in Figure 12. Figure 12 presents the penetration depth as a function of the penetration time, in which *y* is the axial distance and *y* is zero at the bottom of the liner; *t* is the time and *t* is zero when the detonation wave reaches the bottom of the charge; *t*_0_ is the time when the high-density reactive jet reaches the upper surface of the steel target; *τ* is the reactive delay time of the reactive jet; and *H* is the standoff. It should be noted that *L*_max_ is the maximum penetration depth of the corresponding inert jet when the jet tip reaches point *P*, and *L* is the penetration depth of the high-density reactive jet with reaction delay time when the reactive jet reaches point *M* at the time of *τ*. Based on the virtual origin theory [10], the penetration depth of the high-density reactive jet can be expressed as:

(7) $$L=\left(H-a\right)\left[{\left(\frac{\tau -t_{a}}{t_{0}-t_{a}}\right)}^{\frac{1}{1+\sqrt{\rho_{t}/\rho_{j}}}}-1\right]$$

where *ρ*_t_ and *ρ*_j_ are the densities of the steel target and the reactive jet, respectively; (*t*_a_, *a*) is the coordinate of the virtual origin *O*, and its value can be calculated based on the X-ray experiment.

According to Equation (7), it is obvious that the penetration depth of the PTFE/W/Cu/Pb reactive jet is significantly influenced by the standoff and the reaction delay time. Theoretically, reactive materials of the same formula have a constant reaction delay time for a given reactive liner shaped charge. Combing the penetration depth of experimental results with Equation (7), the actual penetration times can be calculated for different standoffs; then we determine that the reaction delay time *τ* of the PTFE/W/Cu/Pb reactive jet is approximately 95.8 μs by averaging the four penetration times. However, when the standoff is 0.5 CD or 2.0 CD, there is a large difference between the actual reaction delay time and the calculated value. In addition to the intrinsic error of the experiments, this is mainly due to the standoff. When the standoff is 0.5 CD, the high-density reactive jet contacts the target at an earlier time with a higher jet tip velocity. Moreover, the diameter of the reactive jet is larger at a smaller standoff, leading to a larger contact area with the target. In fact, these behaviors will cause a higher secondary impact pressure between the reactive jet and the target, which can accelerate the deflagration reaction rate of reactive materials [23,24], thus resulting in a shortened actual reaction delay time. Therefore, the actual reaction delay time of the experiment at 0.5 CD standoff is smaller than the calculated average value. In contrast, the experimental value is larger than the calculated average value for a standoff of 2.0 CD.

**Figure 13:**
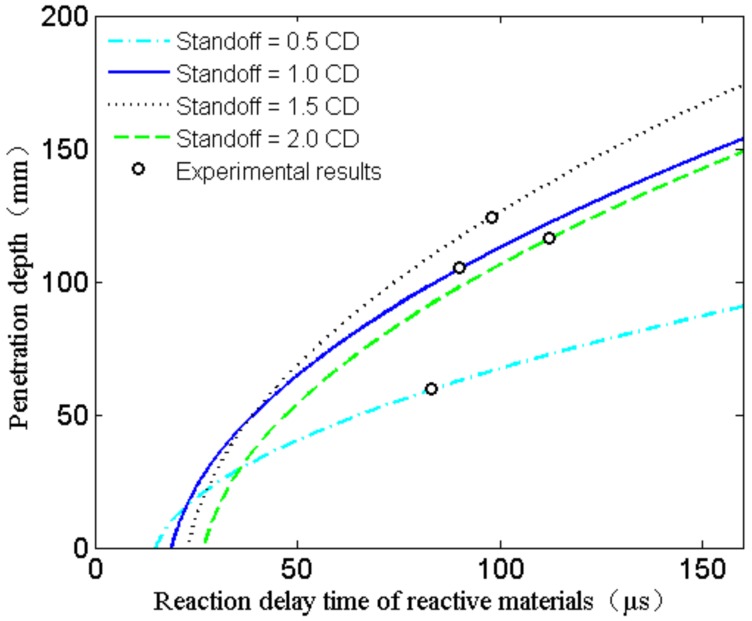
Reaction delay time and standoff affecting the penetration depth.

Based on the experimental results in Section 4.2, the penetration depth of the PTFE/W/Cu/Pb reactive jet against the steel target increases with the standoff, increasing from 0.5 CD to 1.5 CD, which is mainly due to the effective extension of the jet and the long actual reaction delay time (see Figure 13); whereas the penetration depth decreases when the standoff increases from 1.5 CD to 2.0 CD. The main reason is that the PTFE/W/Cu/Pb reactive material liner is a powder material liner in essence, and cavitation or even fractures may occur inside the reactive jet at a larger standoff, thus decreasing the penetration depth of the PTFE/W/Cu/Pb reactive jet.

## 6. Conclusions

The PTFE/W/Cu/Pb high-density reactive material liner was fabricated by the cold pressing and sintering technique. The jet formation and penetration behaviors were studied by X-ray and static experiments. Several conclusions are presented as follows:

(a) The X-ray test results demonstrated that the PTFE/W/Cu/Pb high-density reactive material liner could form a reactive jet with excellent performance under the shaped charge effects, including the distinct jet outline, the clear jet tip and rear, and the satisfied jet continuity.
(b) The results of penetration experiment indicated that, with the standoff increasing from 0.5 CD to 2.0 CD, the penetration depth of the PTFE/W/Cu/Pb reactive jet first increased and then decreased, and the penetration depth could be up to 2.82 CD when the standoff was 1.5 CD. At the same standoff of 1.5 CD, the penetration depth of the PTFE/W/Cu/Pb reactive jet was significantly larger than that of the traditional PTFE/Al reactive jet, but lower than that of the metal copper jet.
(c) The PTFE/W/Cu/Pb high-density reactive jet only occurs with limited and local chemical reactions within the reaction delay time, which indicates that kinetic penetration should be the main defeat mechanism. Once obtaining the reaction delay time, the reactive jet inside the target experiences violent deflagration reactions, releasing large amounts of high-temperature gaseous products, which leads to a sharp pressure rise inside the penetration crater and improves the radial flowing of the target material.

## Figures and Tables

**Table 1:** Experimental results of high-density reactive jets penetrating steel targets.

No.	Standoff (CD)	Penetration Depth (CD)	Penetration Hole Diameter (CD, Charge Diameter)		
			Entrance of ① Plate	Exit of ① Plate	Entrance of ② Plate
#1	0.5	1.36	0.97	-	-
#2	1.0	2.39	0.71	0.24	0.28
#3	1.5	2.82	0.62	0.21	0.24
#4	2.0	2.64	0.55	0.19	0.21

**Table 2:** Comparison of experimental results for different jets.

Materials of Liner	Density (g/cm^3^)	Standoff (CD)	Penetration Depth (CD)	Penetration Hole Diameter (CD)		
				Entrance of ① Plate	Exit of ① Plate	Entrance of ② Plate
PTFE/W/Cu/Pb	8.51	1.5	2.82	0.62	0.21	0.24
PTFE/Al	2.30	1.5	0.70	0.69	-	-
Cu	8.90	1.5	3.85	0.48	0.18	0.21
